# When democracies coerce their health professionals: institutional constraints and policy outcomes across four jurisdictions

**DOI:** 10.3389/fpubh.2026.1878816

**Published:** 2026-09-08

**Authors:** Myeong Su Kim, Daihun Kang

**Affiliations:** 1 Department of Cardiovascular Surgery, Ewha Womans University Seoul Hospital, Seoul, Republic of Korea; 2 College of Medicine, Ewha Womans University, Seoul, Republic of Korea; 3 Department of Plastic and Reconstructive Surgery, Ewha Womans University Seoul Hospital, Seoul, Republic of Korea

**Keywords:** coercive policy, comparative policy analysis, constitutional judicial review, cross-national comparison, health governance, health workforce policy, rule of law

## Abstract

**Background:**

Coercive health workforce policy—the use of legal mechanisms to compel professional compliance in ways that override established processes of negotiation, consent, or collective bargaining—has been deployed repeatedly by governments in high-income democracies through statutory wage controls, contract imposition, employment restructuring, and disciplinary orders backed by license sanctions. The conditions under which such policies achieve or fail to achieve their stated objectives remain poorly understood.

**Methods:**

We conducted a comparative case study of four jurisdictions (South Korea, Ontario/Canada, England, and Hungary) over 2015–2025. Cases were selected from a defined universe of workforce conflicts in high-income democracies to maximize variation in coercive intensity—classified along a five-level escalation ladder derived inductively from the case material—and in institutional configuration, the latter assessed using third-party indices (World Justice Project, Reporters Without Borders, CIVICUS) from editions preceding each conflict’s onset where available. Outcomes were assessed in three layers: compliance with the instrument, persistence of the instrument, and attainment of each government’s stated objective against objective-specific indicators.

**Results:**

In none of the four cases did coercive policy attain its stated workforce objective. South Korea’s return-to-work orders were abandoned amid mass non-compliance, and the quota expansion was reversed during an ensuing constitutional crisis. Ontario’s statutory wage cap was struck down as unconstitutional and repealed. England’s contract imposition produced procedural containment without resolution, followed by training attrition and renewed industrial action. Hungary’s employment restructuring achieved formal compliance amid weakened oversight, yet shortages and emigration persisted. Routine policy correction occurred only where substantive constitutional review of workforce legislation was available (Ontario); where it was absent (England) or eroded (Hungary), coercive policies persisted.

**Conclusion:**

Across these four high-income democracies—including jurisdictions ranking among the top 20 worldwide on rule-of-law indices—coercive workforce policies did not produce the stable, motivated, and adequate health workforces their governments sought, and recourse to compulsion appeared across the democratic spectrum studied. These findings suggest the central policy question is not how to make coercion effective, but how to build the consultation and arbitration mechanisms that make it unnecessary.

## 1 Introduction

A health workforce policy becomes coercive when government employs legal mechanisms to compel professional compliance in ways that override established processes of negotiation, consent, or collective bargaining. Defined in this way, coercion is distinct from the routine unilateralism of everyday policymaking: it is the point at which the state stops persuading its health professionals and starts compelling them. Compulsion has long been recognized as one of the basic families of policy instrument—the “authority tools” that command rather than induce compliance (1), occupying the coercive end of the continuum of governing instruments (2)—and the relationship between the state and the medical profession has been theorized as a contest of countervailing powers in which professional autonomy is periodically curtailed by governmental authority (3). That governments in consolidated democracies—jurisdictions ranking among the top 20 worldwide on international rule-of-law indices (4)—repeatedly resort to such instruments against their own health professionals is itself a finding that warrants examination; that the outcomes of this coercion diverge dramatically across jurisdictions raises a further question about the institutional conditions that shape them. When a government compels rather than persuades its health professionals, under what institutional conditions does that coercion succeed or fail?

Four recent episodes illustrate both the recurrence and the divergence. In February 2024, South Korea’s government announced a 65 percent increase in medical school admissions without consulting the established professional bodies; against a background of long-standing grievances over training conditions, the great majority of trainee physicians submitted their resignations within weeks (5). The government responded with return-to-work orders under the Medical Service Act and license-suspension proceedings against approximately 7,000 physicians; mass non-compliance rendered enforcement impractical, and the quota was ultimately reversed during the caretaker administration that followed the President’s removal, amid a broader constitutional crisis (5). In 2019, Ontario’s provincial government enacted Bill 124, a statutory cap on public-sector wage increases affecting some 780,000 workers including a large share of the health workforce; the law remained in force through the COVID-19 pandemic before being struck down as unconstitutional in 2022 and repealed in full in 2024 (6, 7). In England, after British Medical Association members rejected a renegotiated junior doctors’ contract in a July 2016 referendum (58 to 42 percent), the government imposed the contract from October 2016—following the first all-out physician strike in National Health Service history that April—and the underlying dispute remained unresolved for years (8). In Hungary, parliament passed Act C of 2020 within 2 days in October 2020, restructuring physician employment into a quasi-military status; roughly 95 percent of physicians signed by the March 2021 deadline, yet workforce shortages persisted (9).

These episodes share common features: governments deployed coercive instruments to compel professional compliance with workforce policy objectives, professionals resisted collectively, and outcomes diverged sharply despite superficially similar policy tools—reversal in one setting, prolonged conflict in another, procedural containment without resolution in a third, and formal compliance without substantive success in a fourth. The conflicts also cluster during or immediately after the COVID-19 pandemic; this temporal context is examined descriptively in the analysis but is treated as a background condition that the four-case design cannot control for, an issue we return to in the Discussion (§4.7).

Existing literatures approach this terrain from adjacent directions without addressing it directly. Health workforce research has documented planning, training pipelines, and compensation structures, but has paid less attention to the political dynamics of state–professional confrontation (10); comparative analyses of workforce policy during crisis (11) do not treat coercion as an analytical category. Sriram and colleagues have examined representative health worker organizations as civil society actors in policy processes (12), and Greer and colleagues have analyzed how professional organizations exercise accountability within governance networks, advocating inductive, diagnostic approaches (13). Neither body of work, however, systematically compares the institutional conditions under which coercive workforce policies succeed or fail across jurisdictions—a question of both scholarly and immediate policy significance.

This study addresses that question through a structured comparison of the four cases above, organized around three sub-questions: (a) which coercive instruments governments deployed and what those instruments achieved, judged against each government’s own stated objective; (b) which institutional factors accompanied reversal, modification, or sustained compliance; and (c) how professional collective action interacted with institutional responses. The conflicts examined were predominantly physician–state confrontations—the Ontario case alone extends to nurses and other public-sector health workers—and this physician-weighted scope is treated as a stated condition of the analysis throughout (§2.3, §4.7). The approach is inductive rather than confirmatory: rather than testing a predetermined model, the analysis lets patterns emerge from the cases (13). The aim is not to ask how coercion against health professionals can be made to work, but to identify the conditions under which it fails—and, in doing so, to point toward the negotiated alternatives that might make it unnecessary.

## 2 Methods

### 2.1 Study design

This study employs a multiple case study design with structured cross-national comparison, an established methodology for theory-building in health policy research (14–16). This approach is suited to analyzing complex policy phenomena where causal mechanisms operate through context-dependent pathways and where the limited number of comparable cases precludes large-N statistical analysis (15). The design follows the “building block” approach to cumulative theory development in comparative policy studies (16), combining within-case narrative analysis with structured cross-case comparison to identify patterns inductively.

### 2.2 Key concepts and operational definitions

Two analytical dimensions structure the comparison: the intensity of coercive policy instruments and the strength of institutional constraints. Both are defined operationally to enable independent assessment prior to cross-case comparison. Coercive intensity refers to the degree to which governments employ legal or disciplinary mechanisms to compel professional compliance with workforce policy objectives, bypassing negotiation or consent. Rather than treating coercion as a binary category, this study classifies coercive instruments along a five-level escalation ladder (Table 1), drawing on established distinctions in international labor law (17) and securitization theory (18):

- Level 1—Statutory restriction: Legislative measures that constrain collective bargaining outcomes or professional terms without direct penalties for non-compliance (e.g., statutory wage caps).
- Level 2—Contract imposition: Unilateral implementation of employment terms rejected through negotiation, using employer or legislative authority.
- Level 3—Employment restructuring: Fundamental alteration of professional employment status, imposing new obligations regarding mobility, dual practice, or service conditions.
- Level 4—Coercive deployment: Direct orders compelling specific forms of work, backed by disciplinary sanctions including license suspension or criminal penalties.
- Level 5**—**Securitization: Discursively framing a workforce dispute as a threat to national security or public order, thereby moving it from ordinary politics into the domain of emergency measures. This level draws on the Copenhagen School concept of securitization (18); the instrument is the framing act itself, independent of whether separate emergency powers are invoked for other purposes.

**Table 1:** Analytical dimensions: coercive intensity escalation ladder.

Level	Category	Definition	Case(s)
1	Statutory restriction	Legislative measures constraining collective bargaining outcomes without direct penalties for non-compliance	Ontario (Bill 124)
2	Contract imposition	Unilateral implementation of employment terms rejected through negotiation	England (junior-doctor contract)
3	Employment restructuring	Fundamental alteration of professional employment status, imposing new obligations regarding mobility, dual practice, or service conditions	Hungary (Act C of 2020)
4	Coercive deployment	Direct orders compelling specific forms of work, backed by license suspension or criminal penalties	South Korea (return-to-work orders)
5	Securitization	Discursively framing a workforce dispute as a national-security or public-order threat, moving it into the domain of emergency measures; the instrument is the framing act itself	South Korea (“national health security” framing)

Level 5 draws on the Copenhagen School concept of securitization (18). South Korea’s December 2024 martial-law declaration was directed at the legislature and political opposition, not at health professionals, and is treated as constitutional-crisis context (Table 3), not as a workforce-directed instrument.

This classification distinguishes the policy instruments examined in this study from routine unilateral policy decisions. A policy becomes coercive, as defined here, when it employs legal mechanisms to compel professional compliance in ways that override established processes of negotiation, consent, or collective bargaining. The five levels were derived inductively from the cases examined and are intended as an analytical heuristic rather than a universal taxonomy; boundary cases are discussed in the relevant case narratives. Levels 1–4 escalate in the coerciveness of the instrument deployed; Level 5, securitization, marks a further, discursive escalation—reframing the dispute as a national-security emergency—that may accompany a hard instrument rather than replace it. A case may therefore register a hard-instrument level together with securitization framing, as South Korea did. Securitization was assessed across all four cases and observed only in South Korea.

Institutional constraints refer to the formal and informal mechanisms through which independent actors can review, challenge, or reverse executive action in the domain of workforce policy. This study operationalizes institutional constraint strength through four dimensions, each assessed independently of policy outcomes using established external indicators. These four dimensions correspond to the subfactors of the “Constraints on Government Powers” factor in the World Justice Project (WJP) Rule of Law Index (4), which measures checks on executive authority by the judiciary, legislature, civil society, and the media across 142 countries. The alignment between this study’s analytical dimensions and the WJP framework was identified after the dimensions were derived from the case material, providing independent corroboration of their analytical relevance.

- Constitutional judicial review capacity: The independence and authority of courts to invalidate legislation or executive action on constitutional grounds. Assessed through the WJP Rule of Law Index “Constraints on Government Powers” subfactor on judicial checks and the constitutional provisions for substantive review that exist independently of any given conflict.
- Legislative oversight independence: The capacity of legislative bodies to exercise substantive oversight of executive workforce policy, including committee authority, opposition access, and parliamentary procedures. Assessed through the WJP subfactor on legislative checks and V-Dem (Varieties of Democracy) legislative constraints indicators (19).
- Media plurality: The extent to which independent media can critically report on government workforce policies and amplify professional concerns. Assessed through the Reporters Without Borders World Press Freedom Index (20) and the WJP subfactor on media checks on government.
- Civil society organizational density: The capacity of professional associations, unions, and civil society organizations to mobilize collective action and sustain resistance to coercive policies. Assessed through the WJP subfactor on civil society checks and the CIVICUS Monitor civil society space ratings (21).

Table 2 presents the indicator sources and values for each jurisdiction. The independence of these four dimensions from the policy outcomes is grounded in the provenance of the indicators rather than in a claim of temporal blinding: the World Justice Project, Reporters Without Borders, CIVICUS, and V-Dem indices are produced by third parties for purposes unrelated to these cases, so their values cannot be derived from the outcomes the study seeks to explain. As a further safeguard, for each jurisdiction the index edition closest to**—**and, where available, preceding**—**the onset of the conflict was used, so that the values could not incorporate the conflict’s own institutional consequences; edition years and any post-onset discrepancies are reported in the Table 2 notes. Interventions and mobilization observed during each conflict**—**judicial rulings, legislative responses, and professional collective action**—**are treated as the observed activation pathway (reported separately on the outcome side, Table 3), not as inputs to the ex-ante strength classification. This independence argument is developed further in §2.7.

**Table 2:** Analytical dimensions: institutional constraint dimensions—ex-ante input (external indices only)

Dimension (external index)	South Korea	Ontario/Canada	England/UK	Hungary
Constitutional judicial review (WJP Constraints on Gov. Powers)	~23/142	~10/142	~12/142	122/142
Legislative oversight (WJP + V-Dem)	Strong	Strong	Strong	Weak (supermajority)
Media plurality (RSF, pre-onset ed.; 2024 in parens)	47/180, 2023 (62)	18/180, 2019 (14)	38/180, 2016 (23)	89/180, 2020 (67)
Civil society density (CIVICUS Monitor)	Narrowed	Open	Obstructed (see note)	Obstructed
Ex-ante constraint strength (input only)	Strong	Strong	Strong, with design exception	Weak

WJP, World Justice Project Rule of Law Index, Constraints on Government Powers factor (rank/142). RSF, Reporters Without Borders Press Freedom Index (rank/180); the edition preceding each conflict’s onset is reported, with the 2024 edition in parentheses. CIVICUS ratings: open > narrowed > obstructed > repressed > closed; the UK “Obstructed” rating reflects general civic space, whereas domain-specific professional-association mobilization was high (Supplementary material 1). England “design exception”: under parliamentary sovereignty, primary legislation is not subject to substantive constitutional review—a feature of constitutional design documented independently of the case outcome. Values are third-party indices assessed independently of, and not derived from, this study’s outcomes; the observed activation pathway is reported separately in Table 3.

**Table 3:** Comparative summary of health workforce conflicts across four jurisdictions.

Dimension	South Korea	Ontario (Canada)	England (UK)	Hungary
Coercive intensity level	Level 4 (+L5 framing)	Level 1	Level 2	Level 3
Primary legal instrument	Return-to-work orders; license suspension; “national health security” framing	Bill 124 wage cap (1%/yr., 3 yr)	Contract imposition via NHS employer authority	Act C of 2020 employment restructuring
Governmental framing	National health security emergency	Fiscal sustainability	Service modernization (“seven-day NHS”)	Health system reform; anti-corruption
WJP Rule of Law rank (overall)	19/142	12/142	15/142	73/142
RSF Press Freedom Rank (pre-onset; 2024)	47/180 (62)	18/180 (14)	38/180 (23)	89/180 (67)
CIVICUS civic space	Narrowed	Open	Obstructed	Obstructed
Ex-ante constraint strength (input)	Strong	Strong	Strong, design exception	Weak
Observed activation pathway (outcome)	Non-routine—constitutional crisis → impeachment	Routine—Charter s.2(d) judicial review	Procedural containment—strike rights, no substantive review	None effective
Pandemic context	Post-pandemic; securitization framing	During pandemic; amplified sympathy	Pre-pandemic onset; post-pandemic resurgence	During pandemic; enabled passage
Professional response	Mass resignation (86.7% of trainees)	Multi-union constitutional litigation	Lawful strikes; BMA-led action	Initial opposition; ~95% compliance
Policy outcome	Quota reversed (April 2025)	Bill struck down and repealed (2022–2024)	Contract imposed; unresolved; renewed strikes 2023	Reform implemented; workforce crisis persists

WJP, World Justice Project (overall Rule of Law rank, distinct from the Constraints on Government Powers factor in Table 2); RSF, Reporters Without Borders (pre-onset edition, 2024 in parentheses); NHS, National Health Service; BMA, British Medical Association; Charter s.2(d), Section 2(d) of the Canadian Charter of Rights and Freedoms. Ex-ante constraint strength is the input (Table 2); the observed activation pathway is the result and is not used to define the strength classification.

Professional collective action occupies a defined position in our design. It is a scope condition of the case universe**—**every eligible episode involved collective professional resistance to a coercive instrument**—**and within the analysis we treat it as part of the causal pathway connecting coercive instruments to institutional activation (sub-question c), coded as dimension (c) of the analytical matrix. It is neither an independent explanatory variable nor an outcome.

We define the threshold separating coercion from routine unilateral policymaking by the override of established processes of negotiation, consent, or collective bargaining. The England case marks the lower boundary of the category: no direct compulsion was deployed, but the imposition of employment terms that the profession had explicitly rejected through its negotiated and democratic processes overrides negotiation and consent, and therefore qualifies as coercive under this definition. Unilateral decisions that do not override such processes fall below the threshold and are outside the scope of our study.

#### 2.2.1 Policy outcomes

Outcomes are assessed at three analytically distinct layers, applied uniformly across cases. Layer 1 (compliance)**—**whether the targeted professionals complied with the coercive instrument, observed through participation in or refusal of the mandated action. Layer 2 (instrument persistence)**—**whether the instrument remained in force or was reversed, withdrawn, struck down, or repealed. Layer 3 (objective attainment)**—**whether the policy achieved the government’s own stated objective, evaluated against that objective and a case-specific indicator rather than a common substantive metric. Because the four governments pursued different objectives, no single substantive indicator can measure success across cases; the three-layer structure resolves this, since Layers 1 and 2 are directly comparable across cases while Layer 3 is assessed against each government’s declared goal. “Success” is therefore not treated as a unitary property: a policy may secure compliance (Layer 1) yet fail to attain its objective (Layer 3). Table 4 reports all three layers for each case, with the indicator used for Layer 3. For South Korea, Layer-3 attainment is assessed through the proximate workforce response observable within the study window (residency recruitment); long-run effects on physician supply require follow-up beyond the period examined.

**Table 4:** Stated policy objectives, coercive instruments, and three-layer outcomes.

Dimension	South Korea	Ontario (Canada)	England (UK)	Hungary
Stated policy objective (source)	Expand physician supply; correct regional maldistribution via 65% quota increase (5)	Fiscal restraint in public-sector compensation: 1%/yr. cap, 3 yr. (6)	Workforce reorganization for seven-day NHS services via revised junior-doctor contract (25)	Restructure physician employment; eliminate informal gratuity payments (Act C of 2020) (9)
Coercive instrument (level)	Return-to-work orders + license-suspension (L4); “national health security” framing (L5) (5)	Bill 124 statutory wage restriction (L1) (6)	Contract imposition after membership rejection (L2) (25)	Employment restructuring; sign-or-leave deadline; criminalization of gratuities (L3) (9)
Layer 1—Professional compliance	Mass non-compliance: 86.7% of trainees resigned; 97.3% student boycott; suspension plans withdrawn July 2024 (5)	Formal compliance under statute; sustained multi-union Charter s.2(d) litigation (7)	Lawful strikes incl. First all-out strike (April 2016) (8); terms rejected 58–42; work continued under imposed terms (25)	Formal compliance: ~95–97% signed by March 2021 deadline; 3–5% refused (9)
Layer 2—Instrument persistence	Quota maintained through 2024; reverted for 2026 by the Education Ministry during the caretaker period, April 2025 (5, 32)	In force 2019–2022; struck down Nov 2022; repealed in full Feb 2024 (7)	Imposed Oct 2016; in force throughout the study period (25)	In force from 2021; remained throughout the study period (9)
Layer 3—Stated-objective achievement (indicators)	Not achieved. Quota reversed; 2025 residency recruitment drew applications for 8.7% of first-year positions (314/3,594), obstetrics 0.5% (5, 30)	Not sustained. Restraint held only until invalidation; repeal followed by retroactive awards (7)	Not achieved. Direct specialty-training progression fell from 83 to 42.6% (34); renewed strikes 2023 (35)	Partially/not achieved. High sign rate, but emigration continued and shortages persisted; ~40,000 surgical waitlist by March 2023 (downstream observation) (37)

The three outcome layers are defined in §2.2: Layer 1, professional compliance with the instrument; Layer 2, persistence or reversal of the instrument itself; Layer 3, achievement of the government’s stated objective, assessed against objective-specific indicators. Success and failure are evaluated only at Layer 3, against each government’s own declared aims. Across all four cases Layer 3 was not attained, while Layers 1 and 2 varied. Healthcare-access measures are reported as downstream system observations, not as success criteria. Bracketed numbers refer to the manuscript reference list.

### 2.3 Case selection

#### 2.3.1 Case selection and the case universe

This study employs a diverse-case design (14). The case universe is defined by three scope conditions: (i) a government deployed a coercive workforce instrument—one overriding established processes of negotiation, consent, or collective bargaining (§2.2); (ii) the affected health professionals mounted collective resistance; and (iii) the episode occurred in a high-income democracy in the period surrounding the COVID-19 pandemic. From this universe, four cases were selected to maximize variation on the explanatory dimension—the configuration of institutional constraints—rather than on the outcome: Canada (Ontario) pairs strong constraints with entrenched constitutional review; England pairs strong constraints with the structural absence of substantive review of primary legislation; South Korea pairs strong constraints with activation through a non-routine political pathway; and Hungary represents constraints weakened across all four dimensions. Coercive intensity was permitted to vary across cases (Levels 1–4) so that intensity and constraint configuration could be examined independently.

The four configurations were identified inductively from the case material rather than deduced from a prior model, and the comparison examines how outcomes varied across them rather than testing predetermined predictions. The design nonetheless yields one near-matched contrast that does analytical work: Ontario and England are closely matched on overall rule-of-law strength (WJP Rule of Law Index 12th and 15th of 142) yet differ on a single structural dimension—the availability of substantive constitutional review of primary legislation—allowing the role of that dimension to be examined while other institutional features are held approximately constant. South Korea (WJP 19th) and Hungary (WJP 73rd) extend the range: the former shows strong constraints activating through a non-routine political pathway, the latter shows what follows when constraints are weakened across the board.

Selecting on the explanatory dimension, rather than on observed outcomes, is what distinguishes this design from a selection-on-the-dependent-variable design. Outcomes were assessed only after cases were selected and coded (§2.7), and they vary across all three outcome levels defined in §2.2—which is what permits cross-case comparison.

Two boundaries should be stated plainly. First, scope condition (ii) restricts the universe to episodes in which collective resistance occurred; the study therefore speaks to the trajectory of coercion under professional resistance, not to coercion in general, and cannot characterize cases in which coercive instruments were implemented without resistance or with quiet compliance. Such cases—including coercive measures absorbed within robust corporatist or binding-arbitration frameworks without open conflict—lie outside the universe examined and represent the most important extension of the analysis (§4.2, §4.7). Second, the four cases are illustrative of distinct institutional configurations, not a statistically representative sample; the propositions in §4.4 are offered as hypotheses for testing across further cases, not as estimates generalizable to a defined population.

Our unit of analysis is the jurisdiction holding effective authority over the contested health workforce policy, not the sovereign state. Ontario holds constitutional authority over its public-sector compensation legislation, and England is the NHS jurisdiction within which the contract was imposed; the constitutional constraints relevant to each conflict—the Canadian Charter, the doctrine of parliamentary sovereignty—likewise operate at the level that governs the conflict. What we compare across the four cases is therefore the configuration of workforce-policy authority and the constraints applicable to it. The asymmetry between sovereign and subnational units is noted in the Limitations as a transferability consideration for federal systems with divided authority.

### 2.4 Case descriptions

This subsection sets out the factual background of each case; analytical interpretation is reserved for the Results.

#### 2.4.1 South Korea (2024–2025)

In February 2024 the government announced an increase in annual medical school admissions from 3,058 to 5,058 (65 percent), beginning with the 2025 academic year, citing physician shortages and regional maldistribution, without engaging the established consultation mechanisms with professional bodies (5, 22, 23). Against long-standing grievances over training conditions, 86.7 percent of trainee physicians submitted resignations from 20 February (5), and medical students boycotted classes at a rate of 97.3 percent (5, 24). The government issued return-to-work orders under the Medical Service Act and initiated license-suspension proceedings against approximately 7,000 physicians, framing the walkout as a threat to “national health security” (5).

#### 2.4.2 Ontario, Canada (2019–2024)

In November 2019 the provincial legislature enacted Bill 124, capping public-sector wage increases at 1 percent annually for 3 years across approximately 780,000 workers—a substantial share of them health workers, particularly nurses (6). Multiple unions challenged the statute under section 2(d) of the Canadian Charter of Rights and Freedoms, which protects freedom of association including meaningful collective bargaining (7).

#### 2.4.3 England (2015–2023)

In 2015 the government proposed a revised junior doctors’ contract under its “seven-day NHS” agenda, reducing weekend premium payments while extending standard hours (25). British Medical Association (BMA) members voted 98 percent for industrial action on a 76 percent turnout, culminating in the first all-out strike including emergency care on 26–27 April 2016; a renegotiated agreement endorsed by BMA leadership was rejected by the membership 58 to 42 percent, and the government imposed the contract from October 2016 (8, 25).

#### 2.4.4 Hungary (2020–2023)

In October 2020 parliament passed Act C of 2020 within 2 days, replacing standard physician employment contracts with a new “health service relationship”: an approximately 120 percent salary increase paired with authority for mandatory geographic transfers of up to 2 years, restrictions on dual practice, criminalization of informal gratuity payments, and constraints on mobility and collective representation**—**an employment status comparable to that of the armed forces (9). Physicians faced a March 2021 deadline to sign or leave public employment (9).

### 2.5 Data sources and extraction

Data were drawn from three independent source types. Primary documents—court rulings, legislative texts, parliamentary records, government announcements, and professional-association statements—were obtained from the official repositories of each jurisdiction: the Canadian courts and CanLII for the Bill 124 rulings; BAILII and legislation.gov.uk for the English proceedings and instruments; the Hungarian national legislation repository for Act C of 2020; and Republic of Korea government and National Assembly records for the Korean orders and proclamations. Peer-reviewed literature was identified through systematic searches of PubMed/MEDLINE, Scopus, and Google Scholar, and quality journalism was drawn from national and international news archives.

Search terms combined health workforce, physician strike, medical education reform, back-to-work order, and collective bargaining restriction with each jurisdiction name; the same jurisdiction-paired terms were applied to the peer-reviewed databases and the news archives. Sources were identified through iterative, purposive searching of these repositories, databases, and archives, supplemented by reference chasing from included sources—the identification logic standard to comparative case research, as distinct from the protocol-driven search of a systematic review. The analytical corpus is defined as the full set of cited sources and is inventoried by jurisdiction and source type in Supplementary material 1 (Supplementary Table S1).

Journalistic sources were included only where the outlet had national or international circulation, an established editorial masthead and corrections process, and editorial independence from the government party to the conflict; tabloid, partisan-affiliated, and unattributed sources were excluded.

Extracted information was organized using a standardized analytical matrix comprising six dimensions: (a) coercive instruments deployed, classified according to the escalation ladder; (b) governmental framing and justification; (c) professional collective responses; (d) institutional-constraint activation (courts, legislatures, professional bodies, civil society); (e) pandemic context and its influence on conflict dynamics; and (f) policy outcomes and workforce effects.

Coding of each dimension against the operational definitions, the triangulation of factual claims across source types, and the cross-checking of de facto implementation against the European Observatory on Health Systems and Policies and OECD health workforce reporting are described in §2.7.

### 2.6 Data analysis and synthesis

Analysis proceeded through three phases designed to support inductive pattern identification rather than deductive framework testing (26).

First, within-case analysis constructed detailed narratives for each jurisdiction, tracing the sequence of policy actions, professional responses, and institutional interventions. Each narrative was structured around the six dimensions of the analytical matrix, ensuring comparability across cases while preserving contextual specificity.

Second, cross-case comparison identified patterns of similarity and difference along the two analytical dimensions. Cases were compared pairwise and collectively to assess whether institutional constraint strength (15, 16), independently measured, was systematically associated with policy outcomes. Particular attention was paid to whether similar coercive instruments produced similar or divergent outcomes across different institutional contexts, and to the mechanisms through which institutional constraints operated.

Third, pattern synthesis integrated within-case and cross-case findings to generate analytical propositions about the relationship between coercive intensity, institutional constraints, and policy outcomes. These propositions were assessed against both confirming and disconfirming evidence across the four cases, and anomalies were documented for discussion (27).

### 2.7 Methodological rigor

Several features of the design were intended to support analytical rigor and to keep the inferential chain auditable. The research question, the operational definitions of coercive intensity and institutional constraint, and the case-selection criteria were specified before the detailed within-case analysis, so that the analytical categories were not shaped by the outcomes they were later used to interpret.

The independence of the institutional-constraint measure rests on the provenance of its indicators rather than on a claim of temporal blinding. Constraint strength was assessed from third-party indices—the World Justice Project Rule of Law Index (4), the Reporters Without Borders Press Freedom Index (20), the CIVICUS Monitor (21), and the V-Dem dataset (19)—produced for purposes unrelated to these cases; their values therefore cannot be derived from, or contaminated by, the policy outcomes the study seeks to explain. For each jurisdiction the index edition closest to—and, where available, preceding—the onset of the conflict was used, so that the values could not incorporate the conflict’s own institutional consequences; edition years and any post-onset discrepancies are reported in the Table 2 notes.

The analytical instruments were derived inductively and then checked for external coherence, not imposed *a priori*. The five-level coercive-intensity ladder was abstracted from the four case narratives, and only after the levels were fixed was it compared against established distinctions in international labour law (17) and securitization theory (18). Likewise, the four institutional dimensions emerged from the case material before their correspondence with the WJP “Constraints on Government Powers” subfactors was identified. Stating this sequence explicitly guards against retrospective fitting of the analytical tools to the findings.

Classification against the operational definitions was performed by the lead author. Because the analysis comprises four extensively documented cases rather than a large sample of coded units, an inter-rater agreement coefficient**—**a convention of large-N content analysis**—**is less informative here than full auditability: each classification is justified individually against explicit definitions and named sources. Rigor therefore rests on a complete, inspectable audit trail. The basis and supporting source for every coded value are recorded in a coding rationale log, and the three classifications that did not map cleanly onto a single category**—**the status of the South Korean martial-law declaration, the lower-boundary status of the English contract imposition, and the incentive–coercion hybrid character of the Hungarian reform**—**were adjudicated explicitly against the operational definitions. The coding procedure, the boundary-case adjudications, the source corpus inventory, and the complete log are provided in Supplementary material 1.

Factual claims were triangulated across at least three independent source types**—**primary documents, peer-reviewed literature, and quality journalism**—**and, for non-English jurisdictions, the correspondence between statutory provisions and de facto implementation was cross-verified against the European Observatory on Health Systems and Policies (28) and OECD health workforce reporting (29). Consistent with the logic of comparative case research aimed at theory development, the study prioritizes analytical depth and transferability over statistical generalizability; the propositions in §4.4 are accordingly offered as case-grounded hypotheses for testing rather than as established causal claims.

### 2.8 Use of artificial intelligence

A large language model (Claude, Anthropic) was used, under the authors’ direction, to assist with manuscript drafting and language editing. The study conception, design, data analysis, and interpretation are entirely the authors’ own. All AI-assisted text was critically reviewed, verified against primary sources, and revised by the authors, who take full responsibility for the accuracy, integrity, and originality of the work.

## 3 Results

### 3.1 South Korea: medical school expansion and mass professional exit (coercive intensity: level 4 deployment with level 5 securitization framing)

#### 3.1.1 The workforce dispute

Following the February 2024 announcement (§2.4), the near-total participation in the mass resignation—86.7 percent of trainee physicians (§2.4)—rendered the training system inoperative within weeks (5). The government escalated through two workforce-directed instruments: return-to-work orders under the Medical Service Act backed by license-suspension proceedings against approximately 7,000 physicians (Level 4 coercive deployment), and the discursive framing of the walkout as an emergency threatening “national health security” (Level 5 securitization) (5). Mass non-compliance rendered enforcement impractical: by July 2024, the government withdrew the suspension plans while maintaining the quota expansion (5). Workforce effects were immediate and severe. The 2025 first-half residency recruitment drew 314 applications for 3,594 first-year positions—an application rate of 8.7 percent—with obstetrics receiving one application for 188 positions (0.5 percent) (5, 30).

#### 3.1.2 The constitutional-crisis context and policy reversal

The workforce conflict unfolded alongside, and contributed to the political pressures surrounding, a broader constitutional crisis—including a December 2024 declaration of martial law directed at the legislature and political opposition, reversed by the National Assembly within hours, and the subsequent presidential impeachment and removal (5, 31). The martial-law declaration was not a workforce-directed instrument, and we do not code it as one (§2.7); it is part of the context within which correction ultimately occurred. The quota expansion was reversed for the 2026 academic year in April 2025, the admissions cap restored to its pre-expansion level by the Education Ministry during the caretaker period that followed the President’s removal (5, 32). The case’s analytical significance is precisely this pathway: institutional constraints that were strong by international standards activated not through routine judicial or legislative channels addressed to the workforce policy itself, but through an extraordinary political realignment.

#### 3.1.3 Institutional context

The judiciary maintained formal independence throughout, and the National Assembly’s reversal of martial law within hours demonstrated substantive legislative independence. Media coverage of the conflict remained extensive, and professional associations sustained mobilization despite the absence of formal union or strike rights for physicians. Correction nonetheless occurred through extraordinary political crisis rather than through routine institutional channels.

#### 3.1.4 Pandemic context

The conflict occurred in the aftermath of the COVID-19 pandemic, which had intensified public awareness of healthcare workforce pressures and shaped public sympathy for healthcare workers, while also supplying the government with precedent for framing health workforce issues as matters of national emergency.

### 3.2 Ontario, Canada: wage restraint and constitutional limits (coercive intensity: level 1)

#### 3.2.1 The workforce dispute

Bill 124 represented Level 1 coercion: statutory restriction of collective bargaining outcomes without direct penalties for individual non-compliance. Enacted before the COVID-19 pandemic as fiscal restraint, it remained in force throughout the pandemic, constraining compensation for workers bearing extraordinary burdens. The statute effectively rendered collective bargaining performative—unions could negotiate, but compensation outcomes were predetermined by the cap.

In November 2022, the Ontario Superior Court of Justice struck down Bill 124 as unconstitutional, holding that the 1 percent cap substantially interfered with Charter-protected collective bargaining rights and could not be justified in a free and democratic society (7). In February 2024, the Court of Appeal for Ontario upheld this conclusion in a 2–1 decision; the provincial government declined to seek further review and subsequently repealed Bill 124 in its entirety (7).

#### 3.2.2 Institutional context

Ontario’s institutional constraints were robust. Constitutional judicial review provided an independent mechanism for challenging the legislation, the Canadian Charter offered explicit protection for collective bargaining rights, media coverage documented the impact on healthcare workers extensively, and union organizational infrastructure sustained the legal challenge over nearly 4 years. However, the temporal lag between policy imposition (2019) and final judicial correction (2024) meant that affected workers experienced significant real-wage erosion—compounded by pandemic-era burdens—before institutional correction occurred.

#### 3.2.3 Pandemic context

The COVID-19 pandemic amplified the conflict’s salience. Healthcare workers subject to the wage cap bore extraordinary pandemic burdens, generating substantial public sympathy that strengthened the political and moral case against the legislation. The pandemic did not alter the formal institutional response (judicial review proceeded through established channels), but it likely intensified public pressure on the government’s decision not to appeal further.

### 3.3 England: contract imposition and procedural containment (coercive intensity: level 2)

#### 3.3.1 The workforce dispute

The October 2016 contract imposition (§2.4) represented Level 2 coercion. The government did not deploy the most coercive mechanisms—no back-to-work orders, license suspensions, or criminalization of industrial action. Strike action proceeded through established legal frameworks. Yet the outcome revealed a structural paradox: professionals exercised every institutional tool available to them—collective bargaining, democratic rejection, lawful industrial action, and judicial review—and none proved sufficient to prevent imposition of a contract they had explicitly rejected.

#### 3.3.2 Procedural containment without correction

This paradox is rooted in a distinctive feature of the English constitutional order. Unlike Canada, where the Charter of Rights and Freedoms enabled courts to strike down Bill 124 on substantive constitutional grounds, England has no constitutional court and no mechanism for substantive judicial review of primary legislation. Under the doctrine of parliamentary sovereignty, Acts of Parliament cannot be invalidated on grounds of unfairness, disproportionality, or violation of fundamental labor rights. When a judicial review was brought challenging the contract imposition, the High Court’s inquiry was confined to a single question: had the Health Secretary acted within his statutory powers? The answer was yes, and the review ended there (33). The court did not—and constitutionally could not—evaluate whether the imposed terms were fair, whether they violated collective bargaining principles, or whether they disproportionately burdened a specific professional group. In Ontario, the same type of government action (unilateral override of negotiated terms) was struck down as unconstitutional. In England, it was upheld as lawful—not because the action was substantively different, but because the institutional mechanism to challenge it did not exist.

The consequences of this institutional gap cascaded far beyond the immediate contract dispute. With no constitutional remedy available and the imposed contract in force, junior doctors responded through the only channel remaining: individual exit. The proportion of Foundation Programme doctors progressing directly into specialty training fell from 83 percent in 2010 to 42.6 percent in 2017 (34), as doctors chose career breaks, overseas practice, or alternative careers over continued work under terms they had rejected but could not legally challenge. By 2023—seven years after the imposition—accumulated real-term pay erosion estimated at over 25 percent since 2008 triggered a new wave of industrial action (35), demonstrating that procedural containment had suppressed the conflict without resolving it. The imposed contract did not produce compliance in any meaningful sense; it produced demoralization, attrition, and deferred confrontation.

#### 3.3.3 Institutional context

England’s position in this study is analytically distinctive. Its overall rule-of-law indicators were strong (WJP Rule of Law Index: 15th of 142 countries) (4), and multiple institutional constraints functioned as expected: strike rights were legally protected, industrial action proceeded within established frameworks, and media coverage was extensive. Yet the one institutional mechanism that produced policy correction in the Ontario case—substantive constitutional review of workforce legislation—was structurally absent. England thus represents a natural contrast: what happens when institutional constraints are strong in most dimensions but missing in the specific dimension most relevant to the conflict? The answer, in this case, was procedural containment without substantive correction—an outcome that fell between Ontario’s policy reversal and Hungary’s formal compliance, and that generated long-term workforce deterioration neither side intended.

#### 3.3.4 Pandemic context

The 2016 contract imposition preceded the pandemic, but its downstream effects—workforce demoralization, training attrition, and unresolved pay grievances—were amplified by pandemic-era pressures. The resurgence of industrial action in 2023 occurred in a context where pandemic experience had both increased public appreciation for healthcare workers and normalized emergency-mode policymaking.

### 3.4 Hungary: centralized employment reform under weakened institutional oversight (coercive intensity: level 3)

#### 3.4.1 The workforce dispute

Act C of 2020 represented Level 3 coercion: employment restructuring. The nominal restrictions on dual practice were relaxed through negotiation and weakly enforced, but the mandatory-transfer authority and the constraints on mobility and collective representation fundamentally altered physicians’ employment status. Initial opposition was vocal—thousands publicly stated an intention to refuse—but, facing the sign-or-leave deadline (§2.4), only 3–5 percent ultimately refused (9). This compliance occurred in a context where institutional oversight mechanisms had been significantly weakened: reduced judicial independence, concentrated media ownership, and constrained civil society left physicians without effective channels for redress or collective resistance (36).

#### 3.4.2 Formal compliance without resolution

Formal compliance did not resolve Hungary’s healthcare challenges. Over 8,500 doctors obtained certificates to work abroad over the decade to 2023, and emigration continued (37). By March 2023, approximately 40,000 patients awaited surgical procedures—a historic high; and during the COVID-19 pandemic, Hungarian hospitals recorded among the highest mortality rates per 100,000 population in Europe (37).

#### 3.4.3 Institutional context

Hungary’s institutional constraints had been substantially weakened over the preceding decade. The Orbán government had compromised judicial independence through court-packing and jurisdictional restructuring, collapsed media plurality through state capture and oligarchic consolidation, and constrained civil society organizations through harassment, funding restrictions, and legal obstacles (36). By the time of the 2020 reform, physicians found themselves without effective institutional allies—no independent courts likely to strike down the law, no robust media to amplify concerns, no civil society infrastructure to sustain mobilization. External assessments corroborate this characterization: Hungary’s scores on judicial independence [World Justice Project (4)], press freedom [Reporters Without Borders (20)], and democratic governance [Freedom House (38)] had all declined substantially over the 2010–2020 period.

#### 3.4.4 Pandemic context

The reform was enacted during the COVID-19 pandemic, which provided both context and cover. Pandemic conditions amplified workforce pressures, legitimized governmental claims of emergency, and made collective professional resistance more difficult—physicians were reluctant to withdraw labor during a public health crisis. The pandemic thus functioned as an enabling condition for the reform’s passage and compliance.

### 3.5 Cross-case patterns

Table 3 summarizes key dimensions across the four cases. External indicators of institutional constraint strength, assessed independently of policy outcomes, revealed a clear gradient. Canada ranked 12th globally on the WJP Rule of Law Index (score 0.80) (4), followed by the United Kingdom at 15th (0.78) and South Korea at 19th (0.74)—all within the top quartile of 142 countries assessed. Hungary, by contrast, ranked 73rd (0.51), placing it below the global median and lowest among high-income OECD countries. Press freedom indicators, taken from the RSF edition preceding each conflict’s onset, preserved this ordering: Canada 18th (2019 edition), the United Kingdom 38th (2016), South Korea 47th (2023), and Hungary 89th (2020) (20); the corresponding 2024-edition ranks were 14th, 23rd, 62nd, and 67th. Civil society space ratings diverged further: Canada was rated “Open” by the CIVICUS Monitor—the highest category—while South Korea was rated “Narrowed,” and both the United Kingdom and Hungary were rated “Obstructed,” albeit for different reasons (21).

When these independently assessed institutional indicators were compared against policy outcomes, three cross-case patterns emerged.

**Figure 1:**
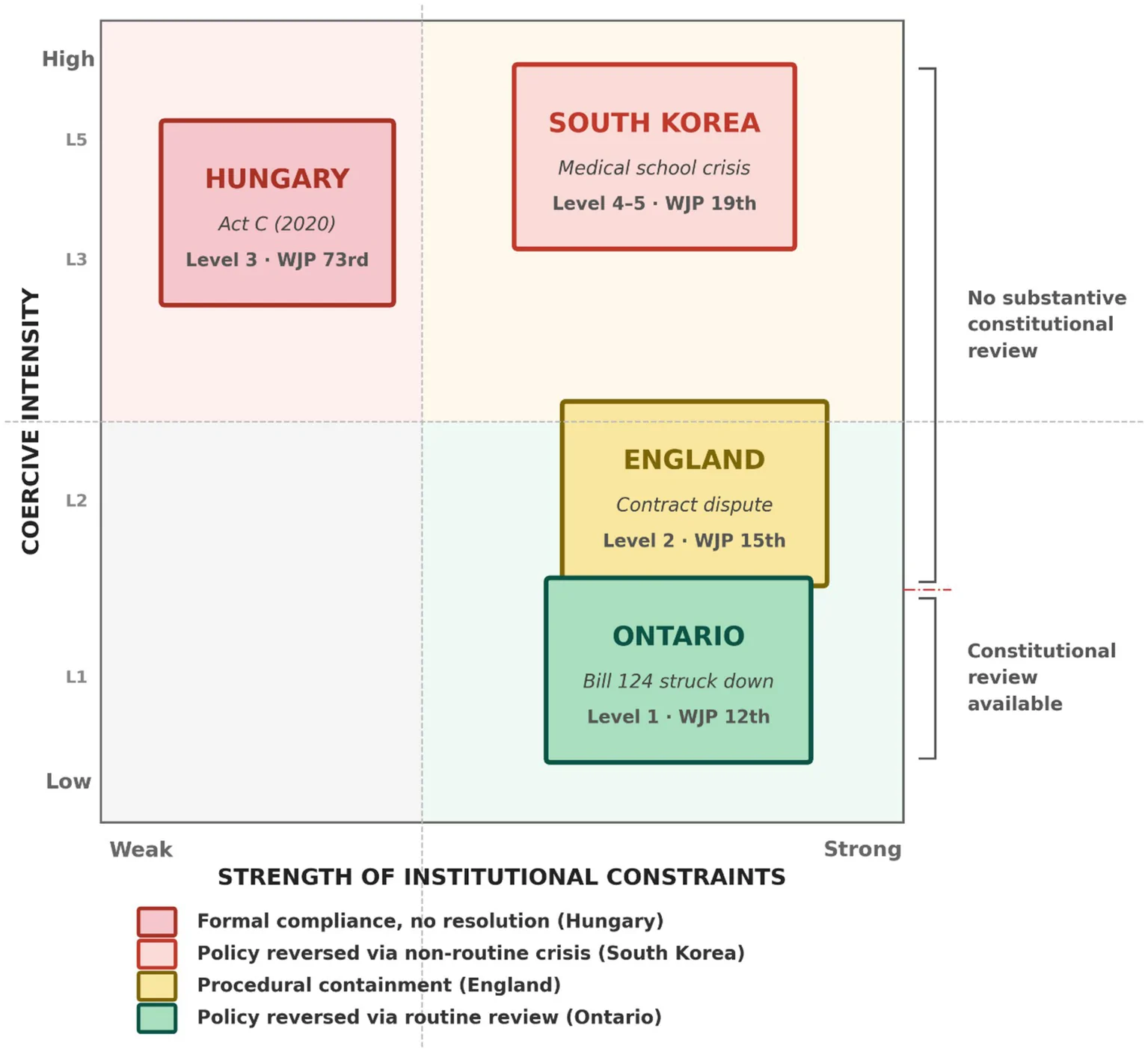
Heuristic positioning of the four cases along two analytical dimensions: coercive intensity (Table 1) on one axis and institutional constraint strength (Table 2) on the other. The placements are schematic and ordinal, intended to summarize the relationships developed in Sections 3–4; they are not quantitative measurements, and the axes carry no metric scale. Positions reflect the analytical coding of each case rather than continuous scores, and inter-case distances should not be read as magnitudes.

*Pattern 1:* Institutional constraint strength was consistently associated with policy outcomes, but the mechanism of institutional activation varied. In both jurisdictions with the strongest overall indicators (Canada, WJP 12th; United Kingdom, WJP 15th), coercive policies were either reversed through routine institutional mechanisms (Ontario: constitutional judicial review) or procedurally contained (England: lawful industrial action). In South Korea (WJP 19th), institutional constraints were comparably strong at baseline, yet activated through an extraordinary pathway—presidential impeachment and constitutional crisis—rather than through routine judicial or legislative channels. In Hungary (WJP 73rd), where all four dimensions of institutional constraint had been substantially weakened, the government achieved formal compliance. This pattern suggests that institutional constraint strength was associated with whether coercive policies were ultimately corrected, while the specific configuration of constraints shaped the pathway through which correction occurred.
*Pattern 2:* Formal compliance did not equate to policy success, and similar overall institutional strength produced divergent outcomes depending on institutional configuration. Even in Hungary—the only case where government achieved formal compliance with restructured employment terms—workforce shortages persisted, emigration continued, and healthcare access deteriorated. Media plurality, measured at each conflict’s onset, differed substantially across the high-coercion pair—South Korea ranked 47th (RSF 2023 edition) against Hungary’s 89th (RSF 2020 edition)—and is therefore consistent with, rather than ruled out as, a contributing condition in that pair. The cleaner isolation of constitutional judicial review comes from the moderate-coercion pair: Ontario and England were closely matched on overall rule-of-law strength (WJP Rule of Law Index, 12th and 15th of 142) and on media plurality at onset (18th and 38th), yet diverged on a single structural dimension—the availability of substantive constitutional review of workforce legislation—and their outcomes diverged accordingly: policy reversal through routine review in Ontario, procedural containment without correction in England. England’s imposed contract remained in force, generating workforce attrition and deferred confrontation rather than institutional correction. Hungary, where both constitutional review and broader institutional constraints had been weakened, achieved formal compliance but no substantive resolution. Figure 1 visualizes the relationship between institutional configuration and observed outcomes across the four cases.
*Pattern 3:* The COVID-19 pandemic functioned as a contextual amplifier rather than an independent causal factor. The pandemic intensified workforce pressures and shaped public framing across all cases, but did not independently determine outcomes. Ontario’s and South Korea’s conflicts were ultimately resolved through pre-existing institutional mechanisms, while Hungary’s outcome reflected institutional weaknesses predating the pandemic. The pandemic amplified existing dynamics rather than creating new causal pathways.

## 4 Discussion

### 4.1 Principal findings

This comparative analysis examined the conditions under which coercive health workforce policies achieve or fail to achieve their stated objectives across four high-income jurisdictions. The central finding is that coercive policy instruments yielded divergent outcomes that varied systematically with the strength of institutional constraints—particularly independent judicial review and constitutional protections for collective bargaining. Coercive policies were ultimately corrected where institutional constraints were strong—through routine constitutional review in Ontario, and, in South Korea, through an extraordinary political realignment rather than routine oversight of the workforce policy itself—procedurally contained but unresolved where review was confined to procedural questions (England), and formally complied with only where institutional constraints had been substantially weakened (Hungary). Even formal compliance, however, did not translate into substantive policy success.

### 4.2 When does coercion succeed? Conditions and limits

The Hungarian case requires careful interpretation. If “success” is defined narrowly as formal compliance with new employment terms, Hungary is the one case that achieved it (§3.4). But the enabling conditions were extraordinary: compliance was secured only after the institutional constraints that might otherwise have channeled resistance—judicial review, media plurality, and civil-society mobilization—had been substantially weakened over the preceding decade. Formal compliance, moreover, did not translate into substantive success: the underlying workforce crisis of shortages, maldistribution, and continued emigration persisted after the reform (§3.4). Governments can compel formal compliance with workforce restructuring, but they cannot compel the motivation, commitment, and retention that functional health systems require.

The temporal dimension further underscores the costs of coercive approaches. Even in jurisdictions with strong institutional constraints, coercive policies imposed significant harm before institutional correction occurred. Ontario’s litigation took nearly four years from enactment to final appellate decision. South Korea’s resolution required extraordinary political crisis. These cases illustrate that institutional constraints do not prevent harm—they eventually correct it, but the lag between policy imposition and institutional response creates substantial interim costs.

These findings should not be overstated, however. The cases examined here capture contexts where coercion met resistance or produced dysfunction, but do not capture configurations where coercion may achieve compliance within robust democracies**—**particularly where binding arbitration mechanisms provide legitimate institutional channels for resolving disputes, or where professional consensus supports policy objectives despite coercive implementation mechanisms. Future research should examine jurisdictions where coercive instruments operated within strong institutional frameworks without triggering the resistance observed in Ontario or England.

### 4.3 The COVID-19 pandemic as contextual amplifier

The temporal clustering of conflicts during or immediately after the COVID-19 pandemic invites consideration of the pandemic’s role. The case comparison revealed that the pandemic operated through three mechanisms—amplifying public sympathy for healthcare workers (Ontario, England), providing enabling conditions for coercive policy passage (Hungary), and supplying securitization framing (South Korea)—but these mechanisms operated in opposing directions. The net effect depended on existing institutional context rather than on the pandemic itself, reinforcing institutional constraints as the central factor shaping outcomes across these cases.

### 4.4 Toward an analytical framework

The cross-case patterns suggest four propositions, offered as empirically grounded hypotheses for examination in other contexts rather than as established causal claims.

*Proposition 1:* Institutional constraint strength was more strongly associated with policy outcomes than coercive intensity. South Korea deployed the highest-intensity coercion yet did not attain its objective; Hungary secured formal compliance with a lower-intensity instrument amid weakened oversight.

*Proposition 2:* Among the institutional constraints examined, the availability of substantive constitutional review was the dimension most closely tied to routine policy correction. The evidential weight rests on the moderate-coercion pair, which most cleanly isolates this dimension: Ontario and England were closely matched on overall rule of law (WJP 12th and 15th of 142) and on press freedom at each conflict’s onset (RSF 18th and 38th), yet diverged on a single structural feature—the availability of substantive constitutional review of workforce legislation—and their outcomes diverged accordingly, routine reversal in Ontario versus procedural containment in England. The same isolation is not available across the high-coercion pair, since South Korea and Hungary differed substantially on press freedom at onset (RSF 47th and 89th); media plurality is therefore consistent with, rather than excluded as, a contributing condition in that pair. On the evidence of these four cases, routine reversal occurred only where substantive constitutional review was available (Ontario); where it was not, coercive policy was not corrected—procedurally contained in England despite otherwise strong rule-of-law indicators, and formally complied with in Hungary amid broadly weakened institutional constraints. The pattern is consistent with constitutional review operating as a last-resort constraint—engaged when negotiation, democratic rejection, and industrial action have all been overridden—though four cases cannot establish this as a general mechanism.

*Proposition 3:* Formal compliance did not translate into substantive workforce outcomes. Hungary’s high compliance coexisted with continued emigration and deteriorating access.

*Proposition 4:* Institutional correction, in every case where it occurred, was delayed, imposing substantial interim harm before it operated.

These propositions can be visualized through a matrix positioning cases along coercive intensity and institutional constraint strength (Figure 1).

### 4.5 Alternative pathways: institutional mechanisms for non-coercive resolution

**Figure 2:**
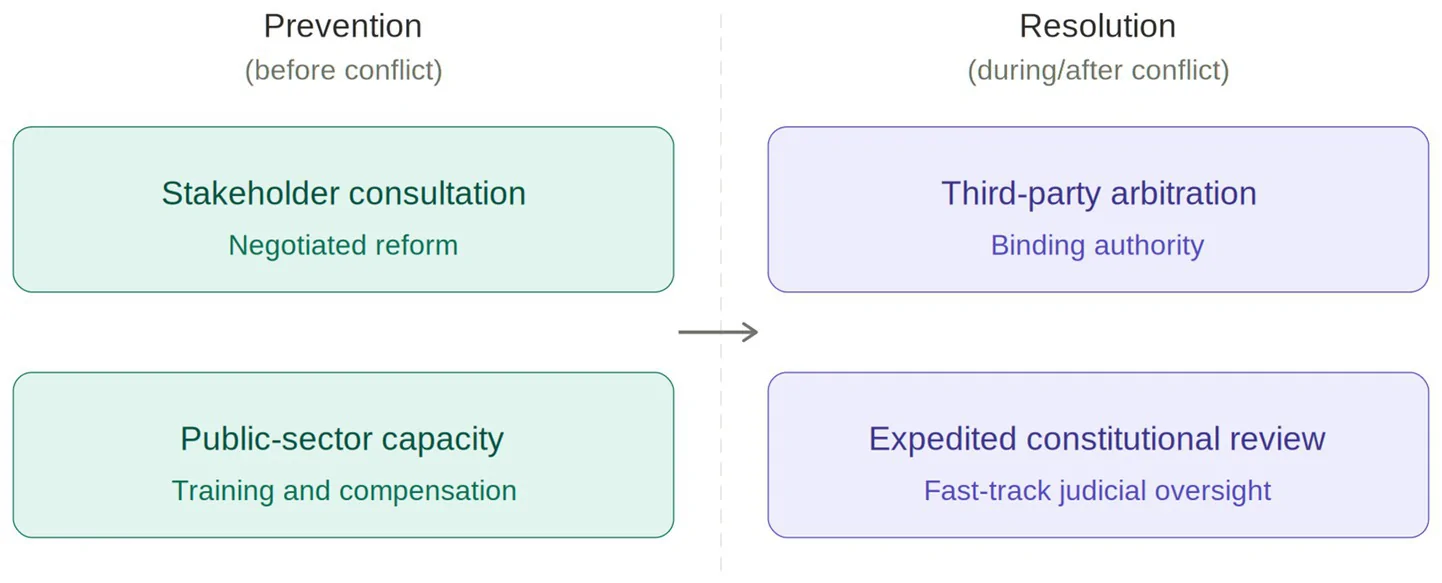
Four institutional mechanisms for non-coercive resolution of health workforce conflicts, derived from cross-case analysis. Prevention mechanisms (left) reduce the need for coercion; Resolution mechanisms (right) constrain coercion when it occurs.

The cases suggest institutional mechanisms that might achieve workforce policy objectives without triggering destructive conflicts (Figure 2).

First, third-party arbitration with binding authority could provide structured channels for resolving disputes before escalation to crisis levels. The absence of such mechanisms in several cases left governments and professionals locked in zero-sum confrontations. Countries with established arbitration frameworks for public-sector labor disputes have generally avoided the acute conflicts observed here.

Second, expedited constitutional review procedures could reduce the temporal lag between potentially unconstitutional policies and judicial correction. Ontario’s years-long litigation illustrates how delayed review allows significant harm to accumulate. England’s absence of any constitutional review mechanism for substantive evaluation of workforce legislation represents a structural gap in institutional protection. Fast-track review mechanisms for legislation affecting fundamental labor rights might provide more timely constraint on governmental overreach.

Third, sustained public-sector workforce capacity through dedicated training pathways, competitive compensation, and sustainable working conditions might reduce governments’ perceived need for coercive measures. South Korea’s crisis arose partly because decades of constraining medical school capacity had created shortages the government sought to address through unilateral expansion rather than negotiated reform (5).

Fourth, institutionalized stakeholder consultation that gives professional organizations meaningful input into workforce policy development might prevent conflicts from reaching crisis levels. Hungary’s reform was enacted within 2 days with no meaningful consultation. South Korea’s quota expansion was announced unilaterally. Jurisdictions with robust corporatist traditions—where professional bodies participate formally in policy development—have generally avoided acute workforce conflicts, though such arrangements carry risks of regulatory capture.

### 4.6 Relationship to existing literature

The patterns identified here are consistent with Sriram et al.’s (12) analysis of professional and health-worker organizations as significant actors in health policy processes. This study extends their work in two directions. First, it shows that professional organizations can influence not only targeted policy decisions but broader governance outcomes, particularly when institutional mechanisms channel professional resistance into constitutional or political correction of coercive policy. Second, it identifies a boundary condition that their framework, centered on organized collective voice, does not address: the form professional resistance takes depends on the legal channels available for it. Sriram et al. (12) theorize health-worker organizations exercising voice—bargaining, advocacy, mobilization—within policy processes; yet in South Korea the capacity for organized labor action was sharply constrained. Health care falls among the essential services in which the right to strike is restricted, and Korean trainee physicians in particular lacked a recognized collective-bargaining agent empowered to call a lawful strike (5). Resistance therefore took the form of mass resignation—an aggregation of individual exits rather than an organized withdrawal of labor, a mode of collective action distinct from a lawful strike (5). This runs counter to a simple capacity-to-resist account: the case in which the organized-voice channel was most constrained (South Korea) produced the most intense resistance (86.7% of trainees), suggesting a non-monotonic relationship between the institutional space for collective voice and the intensity of professional resistance—where voice is foreclosed, exit may substitute, and at greater scale. The intersectional dimensions emphasized by Sriram and colleagues remain important avenues for future investigation that this study does not address in depth (see Limitations).

The inductive analytical approach adopted here aligns with Greer and colleagues’ advocacy for diagnostic governance analysis grounded in empirical complexity rather than simple deductive models (13). The analytical framework proposed in §4.4 is offered in that spirit: as an empirically derived set of propositions, not as a deterministic theory claiming universal applicability.

### 4.7 Limitations

Several limitations warrant acknowledgment.

First, the analysis relies on English-language sources and published documents without primary data collection through expert interviews, meaning that country-specific nuances may be underrepresented. For non-English jurisdictions, analysis depended on official translations and secondary scholarship, with triangulation against European Observatory reports (28) and country-specific academic assessments to verify accuracy.

Second, the study examines four cases from high-income OECD democracies; the analytical propositions may not transfer to middle-income countries, federal systems with divided authority, or contexts where professional organization is weaker. The exclusion of cases where coercive instruments were deployed within strongly institutionalized settings (e.g., jurisdictions with binding arbitration) limits the study’s ability to assess conditions under which coercion might succeed within robust democratic frameworks.

Third, the operational assessment of institutional constraint strength, while grounded in external indicators, involves interpretive judgment. The four-dimensional operationalization improves upon undefined usage but does not eliminate subjectivity. Future research might develop quantitative scoring rubrics to enable more systematic measurement, and the propositions generated here require testing across additional cases and contexts.

Fourth, this analysis does not address intersectional dimensions of workforce conflicts, including gender, class, and inter-occupational power dynamics. The conflicts examined disproportionately involved physicians, whose organizational capacity and market position differ substantially from those of nurses, allied health professionals, and support staff—groups often more affected by coercive workforce policies but less equipped to mount effective collective resistance. Future research examining these intra-professional dynamics would substantially enrich the analytical framework proposed here.

Fifth, several cases occurred during or immediately after the COVID-19 pandemic. While the pandemic’s role is analyzed as a contextual amplifier, the temporal clustering limits the study’s ability to disentangle pandemic-specific dynamics from broader patterns of state–professional conflict.

## 5 Conclusion

Across four high-income democracies, coercive health workforce policies did not achieve their stated objectives in any of the cases examined. Where institutional constraints were strong, coercive policies were reversed or contained; where constraints were weak, governments secured formal compliance—but workforce shortages persisted, emigration continued, and healthcare access deteriorated. In none of the four cases did coercion produce the outcome policymakers sought: a stable, motivated, and adequate health workforce.

Among the cases examined, the institutional constraint most closely tied to outcomes was substantive constitutional review of workforce legislation—the only dimension whose presence coincided with routine policy correction. Yet even where it was available, it did not prevent coercion from being attempted; it operated only after the fact, ensuring eventual reversal at the cost of substantial interim harm. And where this mechanism was structurally absent, as in England, even a democracy ranking 15th globally in rule of law could not prevent imposition of a contract its own professionals had democratically rejected. These patterns emerge from cases selected to vary on institutional configuration rather than on their outcomes; they are therefore best understood as hypotheses the comparison generates for testing elsewhere, not as causal effects estimated from a representative sample.

Perhaps the most sobering observation is that every jurisdiction examined—including consolidated democracies ranking among the top 20 worldwide—resorted to coercion when faced with workforce pressures. The temptation to compel rather than negotiate was not confined to weakened democracies; it recurred across the democratic spectrum of these cases. For policymakers, the question these cases pose is not how to make coercion effective, but how to build the consultation and arbitration mechanisms that make it unnecessary.

## Data Availability

Publicly available datasets were analyzed in this study. This data can be found at: The following publicly available datasets and indices were analyzed in this study. None of these resources use accession numbers; all are accessible through public web portals. 1. World Justice Project Rule of Law Index Repository: World Justice Project URL: https://worldjusticeproject.org/rule-of-law-index/ Accession number: Not applicable (publicly accessible index). 2. Reporters Without Borders World Press Freedom Index Repository: Reporters Without Borders (RSF) URL: https://rsf.org/en/index Accession number: Not applicable. 3. CIVICUS Monitor Repository: CIVICUS World Alliance for Citizen Participation URL: https://monitor.civicus.org/ Accession number: Not applicable. 4. V-Dem (Varieties of Democracy) Dataset Repository: V-Dem Institute, University of Gothenburg URL: https://v-dem.net/data/the-v-dem-dataset/ Accession number: Not applicable. 5. OECD Health Statistics Repository: OECD.Stat URL: https://stats.oecd.org/ Accession number: Not applicable. All additional primary documents analyzed in this study (court rulings, parliamentary records, government documents, professional association publications, and quality journalism) are cited in the References section of the manuscript and accessible through the citations provided.
