# Supplementary material for: When democracies coerce their health professionals: institutional constraints and policy outcomes across four jurisdictions
Source: Front Public Health. 2026 Sep 8;14:1878816. doi: 10.3389/fpubh.2026.1878816 (PMC13593763; doi:10.3389/fpubh.2026.1878816)
Supplement: Supplementary file 1 [file Data_Sheet_1.docx]

**Supplementary Material 1**

**Coding Procedure, Boundary-Case Adjudications, and Audit Trail**

*When Democracies Coerce Their Health Professionals: Institutional Constraints and Policy Outcomes Across Four Jurisdictions*

## 1. Coding Procedure

Extracted information was organized using the six-dimension analytical matrix described in the main-text Methods (coercive instruments deployed; governmental framing; professional collective responses; institutional-constraint activation; pandemic context; policy outcomes and workforce effects). Classification against the operational definitions was performed by the lead author, and the basis and supporting source for each classification were recorded in the coding rationale log (Table S2) to permit independent verification. Because the analysis comprises four extensively documented cases rather than a large sample of coded units, an inter-rater agreement coefficient — a convention of large-N content analysis — is less informative here than full auditability; transparency rests instead on the documented rationale and source for every coded value.

Classifications that did not map cleanly onto a single category were flagged and adjudicated against the operational definitions; these boundary cases are set out in Section 2 and recorded in Table S2. The final manuscript, including these adjudications, was reviewed and approved by both authors. The analytical corpus — defined as the full set of cited sources — is inventoried by jurisdiction and source type in Table S1.

## 2. Boundary-Case Adjudications

### 2.1 South Korea — martial law treated as context, not a workforce instrument

South Korea’s coercive instruments were classified at Level 4 (return-to-work orders under the Medical Service Act, backed by license-suspension proceedings against approximately 7,000 physicians) together with Level 5 securitization, the latter referring specifically to the government’s framing of the walkout as a “national health security” emergency. The declaration of martial law in December 2024, although temporally proximate, was directed at the National Assembly and the political opposition rather than at health professionals; it is therefore treated as part of the broader constitutional-crisis context rather than as a workforce-directed coercive instrument. This distinction prevents two temporally and functionally distinct state actions from being bundled into a single intensity assessment, and clarifies that the eventual correction operated through an extraordinary political pathway rather than through constitutional judicial review of the workforce policy.

### 2.2 England — the lower-boundary case for the definition of coercion

England is the threshold case for the operational definition of coercion. The government deployed no return-to-work orders, license suspensions, or criminalization of industrial action, and strike action proceeded lawfully. The contract imposition nonetheless meets the definition because it implemented employment terms that had been explicitly rejected through the profession’s negotiated and democratic processes (British Medical Association membership rejection, 58%–42%) using employer authority — thereby overriding established processes of negotiation and consent. It is classified at Level 2 (contract imposition) and identified as the case defining the lower boundary of the coercive category, distinguishing coercive instruments from routine unilateral policy decisions.

**Civil-society dimension.** The CIVICUS Monitor rating of “Obstructed” for the United Kingdom reflects general civic-space conditions and is retained as the external value in the main-text institutional table. In the specific domain of professional-association capacity, mobilization was high (a 98% strike ballot and the first all-out NHS physician strike). This divergence between the general external index and the domain-specific observation is recorded rather than reconciled; the external index value is not overridden, and England’s overall classification as institutionally strong rests on its judicial, legislative, and media dimensions independently of the civil-society entry.

### 2.3 Hungary — incentive–coercion hybrid and media triangulation

Hungary’s Act C of 2020 is classified at Level 3 (employment restructuring). The reform paired a substantial salary increase (approximately 120%) with restriction of professional freedoms, a sign-or-leave deadline, mandatory geographic transfers of up to two years, and criminalization of informal gratuity payments. The presence of a financial inducement does not remove the coercive character of the instrument: the compulsory alteration of employment status, the ultimatum to accept or exit public employment, and the criminal sanction together satisfy the operational definition. The criminalization element targets gratuity payments rather than the refusal to work, and therefore does not raise the classification to Level 4 (which concerns direct orders compelling work).

**Media-plurality dimension.** At conflict onset, Hungary’s pre-onset RSF rank (89/180, 2020 edition) was substantially weaker than South Korea’s pre-onset rank (47/180, 2023 edition), directly consistent with the documentary record of state capture and oligarchic consolidation of ownership. The apparent convergence of the 2024-edition ranks (67 vs 62) post-dates both conflicts and reflects South Korea’s subsequent decline rather than Hungarian improvement at the time of Act C. Hungary’s media-plurality dimension is therefore coded as substantially weakened on the basis of both the pre-onset index and the qualitative record.

## 3. Table S1 — Source Corpus Inventory

*Cells list the citation numbers in each source-type × jurisdiction bucket. Counts reflect the numbered reference list ([1 - 38]); the column totals sum to 38.*

| **Source type** | **South Korea** | **Ontario / Canada** | **England / UK** | **Hungary** | **Cross-national** |
| --- | --- | --- | --- | --- | --- |
| **Primary legal / official documents** | — | — | [33] | — | [17] |
| **Peer-reviewed literature** | [5, 24] | — | [8, 34] | [9, 36] | [1, 10, 11, 12, 14, 26] |
| **Scholarly books (theory & methods)** | — | — | — | — | [2, 3, 13, 15, 16, 18, 27] |
| **Quality journalism** | [22, 23, 30, 31, 32] | [6, 7] | [25, 35] | [37] | — |
| **Institutional indices & reports** | — | — | — | [28] | [4, 19, 20, 21, 29, 38] |
| **Total (numbered sources)** | 7 | 2 | 5 | 4 | 20 — Total 38 |

***Note.*** *“Cross-national” covers theory, methods, and third-party indices not specific to one jurisdiction. Primary legal instruments — the Korean Medical Service Act and return-to-work orders (Republic of Korea government and National Assembly records); Ontario Bill 124 and the 2022 Superior Court and 2024 Court of Appeal rulings (CanLII); the imposed junior-doctor contract and related instruments (legislation.gov.uk), together with the High Court ruling [33]; and Act C of 2020 (Hungarian national legislation repository) — were consulted from official repositories (§2.5) and are cited in-text; only separately numbered sources are tabulated above.*

## 4. Table S2 — Coding Rationale Log (Audit Trail)

*Each coded value is accompanied by the basis for the classification and its supporting source(s), permitting independent verification. Bracketed numbers refer to the manuscript reference list.*

| **Case** | **Coding element** | **Classification** | **Basis** | **Source** |
| --- | --- | --- | --- | --- |
| **South Korea** | Coercive intensity | Level 4 + Level 5 (framing); martial law = context | Return-to-work orders + license-suspension proceedings = Level 4; “national health security” framing = Level 5. Martial law directed at the legislature/opposition, not at professionals → excluded as a workforce instrument. | [5] |
| **South Korea** | Institutional strength | Strong | Top-quartile WJP; legislative independence evidenced by the National Assembly’s reversal of martial law; press and professional mobilization active. | [4][5][19][20][21] |
| **Ontario** | Coercive intensity | Level 1 | Statutory wage cap (Bill 124) constraining collective-bargaining outcomes without penalties for individual non-compliance — clean fit to Level 1. | [6] |
| **Ontario** | Institutional strength | Strong | Top-decile WJP; substantive constitutional review of legislation entrenched via the Charter; CIVICUS “Open”; sustained multi-union litigation. | [4][7][19][20][21] |
| **England** | Coercive intensity | Level 2 (lower-boundary case) | Imposition of a democratically rejected contract via employer authority; no direct compulsion (no return-to-work orders or license suspensions). Defines the boundary between coercion and unilateral policy. | [8, 25,33] |
| **England** | Institutional strength | Strong, design exception | WJP 15th overall, but no substantive review of primary legislation under parliamentary sovereignty — a design feature documented independently of the case outcome. CIVICUS “Obstructed” (general civic space) is retained as the external value; the divergence from domain-specific professional-association mobilization is recorded without overriding the index. | [4, 19, 20, 21, 33] |
| **Hungary** | Coercive intensity | Level 3 (incentive–coercion hybrid) | Employment restructuring with sign-or-leave ultimatum, mandatory transfers, and criminalization of gratuities. Pay inducement does not negate coercion. Criminalization targets gratuities, not refusal to work → not Level 4. | [9] |
| **Hungary** | Institutional strength | Weak | All four dimensions eroded over the preceding decade (WJP overall 73rd, Constraints on Gov. Powers 122nd). Media coded weak via triangulation despite RSF 67/180 (2024 ed.), on the basis of the pre-onset rank (89/180, 2020 ed.) and documented state capture. | [4, 19, 20, 36, 38] |

**Supplementary Figure S1.** Per-case timelines of the four health workforce conflicts, 2015–2025. Colored bars indicate the period during which each coercive instrument was in force; circles mark key events; upward triangles (▲) mark professional collective action and industrial-action events; inverted triangles (▼) mark downstream workforce or system observations; diamonds mark institutional responses. The shaded band denotes the COVID-19 Public Health Emergency of International Concern period. For South Korea, the December 2024 martial-law declaration is shown as a dashed contextual marker rather than an instrument bar, reflecting its classification as part of the broader constitutional crisis rather than a workforce-directed coercive instrument (§2.5; Supplementary Table S2). Outcome labels use the same color scheme as Figure 1.
